# Complete mucosal healing prevents stricture progression after endoscopic balloon dilation in Crohn's disease

**DOI:** 10.1002/deo2.70121

**Published:** 2025-04-23

**Authors:** Jun Owada, Kunihiko Oguro, Tomonori Yano, Yusuke Ono, Takuma Kobayashi, Shoko Miyahara, Hirotsugu Sakamoto, Hironori Yamamoto

**Affiliations:** ^1^ Department of Medicine Division of Gastroenterology Jichi Medical University Tochigi Japan

**Keywords:** balloon dilation, Crohn's disease, endoscopy, intestinal strictures, restenosis

## Abstract

**Objectives:**

Endoscopic balloon dilation (EBD) is an effective treatment for intestinal strictures in Crohn's disease (CD). However, restenosis often occurs and requires repeat EBD or surgery. Previous studies have seldom examined restenosis with respect to stricture diameter, leaving the factors contributing to post‐EBD restenosis unclear. Our retrospective study indicated that complete mucosal healing significantly reduces restenosis after EBD in CD‐related small intestinal strictures. This prospective study aimed to validate these findings by accurately measuring stricture diameters in patients with CD.

**Methods:**

We conducted a single‐center prospective study of patients with CD and small intestinal strictures. The patients underwent an EBD session between June 2022 and December 2023. Stricture diameters were measured using a calibrated small‐caliber‐tip transparent hood. Multivariate analysis was performed to identify factors influencing stricture progression.

**Results:**

This study included 41 patients (33 men). The number of strictures detected between sessions increased from 159 to 170. The average diameter of all strictures and the narrowest stricture per patient showed slight increases. However, 73% of patients experienced stricture progression. The presence of ulcers between sessions was identified as a significant risk factor for stricture progression (odds ratio 7.59, *p* = 0.031). Patients achieving complete mucosal healing demonstrated a significant increase in the narrowest stricture diameter (+1.5 mm, *p* = 0.00089).

**Conclusions:**

Complete mucosal healing is crucial for preventing stricture progression after EBD in patients with CD‐related small intestinal strictures.

## INTRODUCTION

Crohn's disease (CD) is a chronic inflammatory bowel disease characterized by alternating remission and relapse. A significant complication is fibrotic intestinal strictures,^1^, ^2^ observed to increase from 11%–14% to 21%–35% within 5 years post‐diagnosis despite treatment advances.^3^, ^4^ Symptomatic small intestinal strictures often require surgery,^4^ with 24.2% needing a second surgery within 5 years,^5^ although repeated surgeries risk short bowel syndrome. Consequently, endoscopic balloon dilation (EBD) for short, uncomplicated strictures (<5 cm) has gained favor.^6^ EBD is less invasive and generally safe,^7^, ^8^ though restenosis frequently necessitates further interventions.^9^ Long‐term outcomes, measured by symptom‐free and surgery‐free survival,^10^, ^11^, ^12^ are influenced by factors such as diet and surgical decisions. Therefore, an objective evaluation of stricture diameters is essential, but this is not routinely performed.

At our institution, we use a calibrated small‐caliber‐tip transparent (CAST) hood (TOP Corporation) during double‐balloon enteroscopy (DBE) with EBD to accurately measure stricture diameters. Previous research suggested that complete mucosal healing is crucial for preventing restenosis in CD‐related strictures.^13^ However, that study had limitations, including a small cohort, absence of quality of life (QOL) metrics, and omission of new biomarkers like leucine‐rich alpha‐2 glycoprotein (LRG).^14^ This prospective study further assesses EBD effectiveness for small intestinal strictures in CD, focusing on stricture progression and risk factors via precise diameter measurements.

## METHODS

### Patients

At our institution, most patients with CD undergo annual DBE to monitor and optimize treatment. A proactive treat‐to‐target approach was adopted, scheduling DBE with EBD for symptomatic and asymptomatic patients. This strategy advocates endoscopic evaluation, even in the absence of symptoms, to comprehensively assess disease activity. If endoscopic findings reveal disease activity despite normal laboratory results and the absence of symptoms, patients are advised to adjust their medication regimen to better manage CD. EBD is performed when strictures are identified, allowing endoscope passage and examination of additional intestinal segments. Total enteroscopy was performed only when necessary, after carefully considering the balance between risks and benefits. Because lesions in most patients with CD occur in the distal ileum, total enteroscopy is required in approximately 5%–10% of cases. When no additional strictures were observed for approximately 50 cm, we employed selective contrast imaging to evaluate the deep sections of the small intestine for any additional strictures.

This prospective cohort study initially enrolled 94 patients with CD who underwent DBE with EBD between June 2022 and December 2023. The inclusion criteria for this study were as follows: (1) a diagnosis of ileal or ileocolonic CD,^15^ (2) de novo strictures in the small intestine meeting EBD criteria, (3) a scheduled EBD procedure, and (4) consent to participate in the study. Anastomotic strictures were excluded because their characteristics differ from those of *de novo* small intestinal strictures, as the patterns of stricture formation and ulceration may vary depending on surgical and anastomotic techniques. EBD was performed for short fibrous strictures (<5 cm) that obstructed the endoscope or overtube passage regardless of symptoms. EBD was contraindicated for strictures with deep ulcers, fistulas, severe angulation, or abscesses. Patients with incomplete data were excluded from the analysis. The Institutional Ethical Review Board of Jichi Medical University approved this study, which was conducted in accordance with the principles outlined in the Declaration of Helsinki.

### Sessions and procedures

A series of DBE procedures performed during a single hospitalization was classified as “one DBE session.” In one DBE session, retrograde, antegrade, or both approaches of DBE were performed as necessary. We aimed to perform sequential EBD for all identified strictures. During the EBD and follow‐up sessions, all detected strictures were assessed using DBE and treated with EBD as necessary. Prior to the procedure, the patients provided written informed consent for DBE, EBD, conscious sedation, and study participation.

#### Double‐balloon enteroscopy

Ulcers and strictures were evaluated using therapeutic DBE (EN‐580T, EI‐580BT, or EN‐840T; Fujifilm Corporation) with an overtube (TS‐13140 or TS‐13101; Fujifilm Corporation). Ulcers were defined as mucosal defects of any size or depth, including small lesions. Strictures were identified as luminal narrowing with an internal diameter of ≤15 mm based on the endoscope or overtube passage.

#### Measuring stricture diameter

**FIGURE 1:**
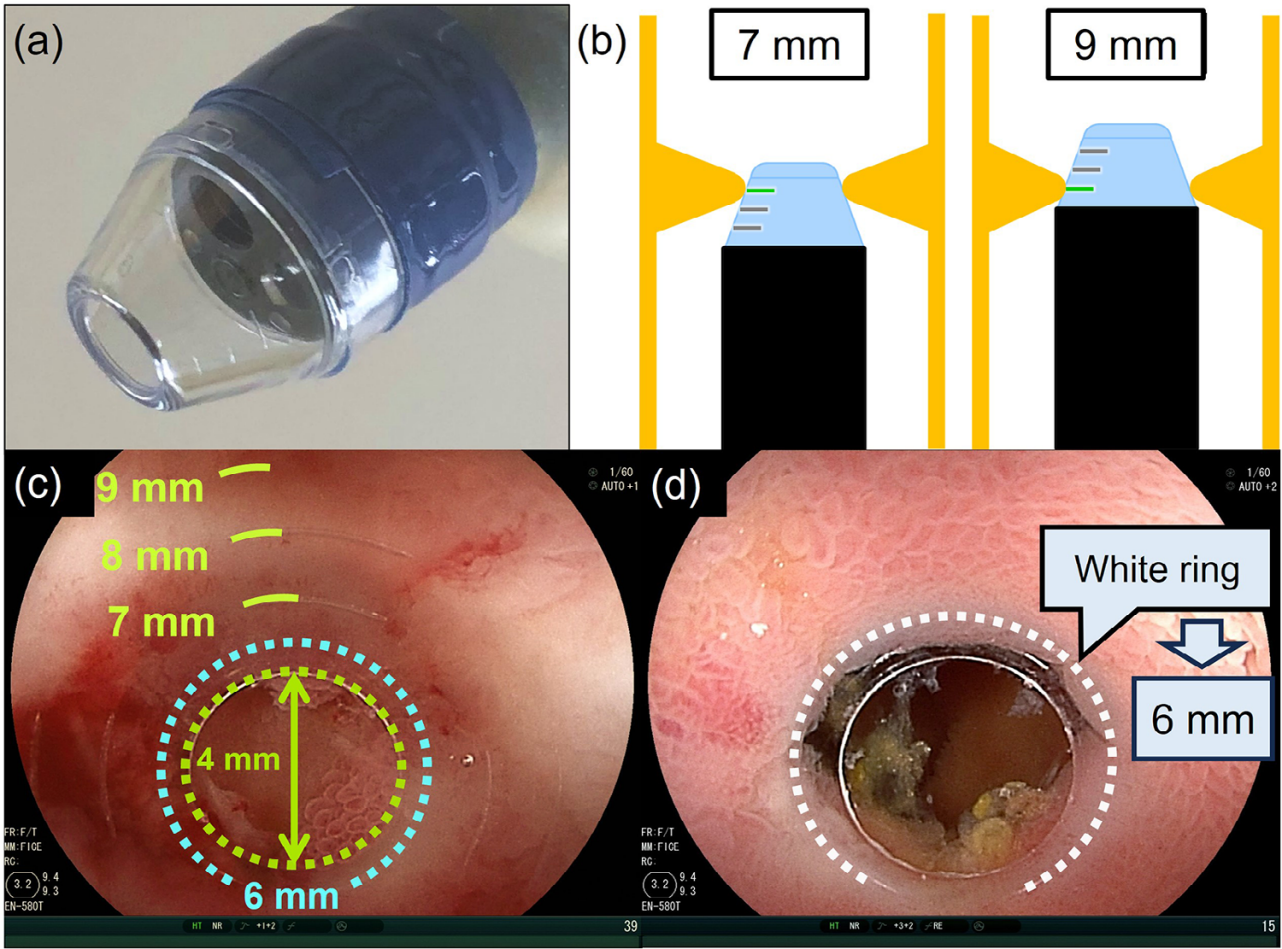
Calibrated small‐caliber‐tip transparent (CAST) hood and illustrative images of small intestine strictures. (a) The CAST hood attached to the endoscope tip. (b) Measurement of the inner diameter of the stricture by positioning the CAST hood within it. (c) Green‐highlighted calibration lines on the CAST hood, with a white ring indicating the tightest part of the stricture. The measured inner diameter is 8 mm. (d) A severe stricture without ulceration (6 mm diameter).

Stricture diameters were measured using a CAST hood, designed for sequential EBD and precise stricture measurements (Figure 1).^16^, ^17^ The CAST hood features calibration lines (7, 8, and 9 mm) and landmarks (orifice: 4 mm, edge: 6 mm, and outer ring: 10 mm), aiding objective measurement by identifying a “white ring,” visible upon careful insertion into the stricture, indicating the fibrotic portion (Figure 1c).^16^, ^17^ For mild strictures, diameters were estimated based on the resistance encountered by the CAST hood, attached to the endoscope (11 mm outer diameter) and overtube (13.2 mm outer diameter), when passing through the stricture (Table 1). Strictures were classified based on their diameter as mild (11 mm or more), moderate (8–10 mm), or severe (≤7 mm).

**TABLE 1:** Techniques for measuring stricture diameters with the CAST hood in double‐balloon enteroscopy.

Stricture diameter	Methods to describe the stricture diameter
4 mm	The white ring is the same as the opening of the CAST hood.
5 mm	The white ring is larger than the opening but less than the edge of the CAST hood.
6 mm	The white ring is the same as the edge of the CAST hood.
7–9 mm	The calibration lines of the CAST hood at the white ring
10 mm	Scope passing with a certain resistance with the CAST hood
11 mm	Scope passing with slight resistance with the CAST hood
12 mm	Scope passing without resistance with the CAST hood
13 mm	Overtube passing with a certain resistance
14 mm	Overtube passing with slight resistance
15 mm	Overtube passing without resistance

Abbreviation: CAST, calibrated small‐caliber‐tip transparent.

#### Endoscopic balloon dilation

After measuring the stricture diameter, an endoscopic guidewire (RevoWave; Piolax or Jagwire Plus; Boston Scientific) and a through‐the‐scope balloon catheter (CRE Pro GI Wire guided; Boston Scientific or Elation 3‐stage Wire guided Dilation Balloon; Merit Medical Systems) were advanced through the stricture lumen and inflated to the target diameter under fluoroscopic guidance. The balloon was maintained at the target pressure for 1 min. In cases with multiple strictures, each stricture was measured and treated with EBD. EBD is not always necessary for mild strictures to allow the endoscope to pass; however, it is sometimes performed to enable the overtube to pass, depending on the clinical situation. The choice of balloon catheter size was guided by the internal diameter of the stricture: an 8–10 mm catheter for strictures of 4 mm or less, a 10–12 mm catheter for strictures of 4–7 mm, and a 12–15 mm catheter for strictures with a diameter >7 mm. EBD is generally performed at the same diameter regardless of the presence of ulcers. However, due to the increased risk of bleeding and perforation in ulcerative strictures, we started with a small diameter and adopted a stepwise approach. The final balloon size was selected after considering factors such as ulcers, inflammation degree, stricture diameter and length, and the need for an endoscope or overtube passage. The target diameter of EBD was carefully adjusted to balance the associated risks and benefits. Endoscopic passage through the stricture was confirmed after each EBD procedure.

### Outcomes analysis

The primary outcome was the change in stricture diameter, which was used to evaluate the effectiveness of a single EBD session. Secondary outcomes included the rates of stricture progression or improvement, frequency of procedure‐related adverse events, accuracy of biomarkers such as LRG, and changes in the CD activity index (CDAI) and short inflammatory bowel disease questionnaire (SIBDQ). The CDAI is a clinical tool for the accurate, objective, and reproducible assessment of disease activity in patients with CD.^18^, ^19^ Additionally, the SIBDQ was shown to be valid, reliable, and capable of detecting meaningful changes in quality of life,^20^ licensed by Dr. Jan Irvine et al. from McMaster University, Canada.

We compared endoscopic findings, including the frequency and diameter of strictures, from EBD and follow‐up sessions. Because most patients had multiple strictures, some resolved while new ones developed, we focused on the narrowest stricture diameter per session for each patient to evaluate stricture progression and improvement post‐EBD. Our approach emphasized gradual, stepwise dilation rather than achieving maximal dilation in a single session. While endoscopic passage was confirmed post‐dilation, restenosis was frequently observed at the 1‐year follow‐up. We defined progression as a change of +1 mm or less in the narrowest stricture diameter, whereas an increase of +2 mm or more was considered an improvement, indicating a favorable clinical course. Based on these criteria, the patients were divided into two groups: those with progression and those showing improvement. The analysis focused on identifying factors associated with progression after EBD.

### Statistical analysis

All statistical analyses were conducted using EZR software, version 1.61 (Jichi Medical University Saitama Medical Center).^21^ Fisher's exact test was used for categorical variables, whereas Student's t‐test or the Mann–Whitney U test was used for quantitative variables. Paired t‐tests were used to assess changes in the narrowest stricture diameters between sessions. The Tukey's test was used for multiple group comparisons. Logistic regression analysis identified factors associated with progression. Receiver operating characteristic (ROC) analysis was used to determine the optimal biomarker cut‐off values, with sensitivity, specificity, and area under the curve (AUC) calculated. A *p*‐value < 0.05 was considered statistically significant.

## RESULTS

### Background characteristics of patients

**FIGURE 2:**
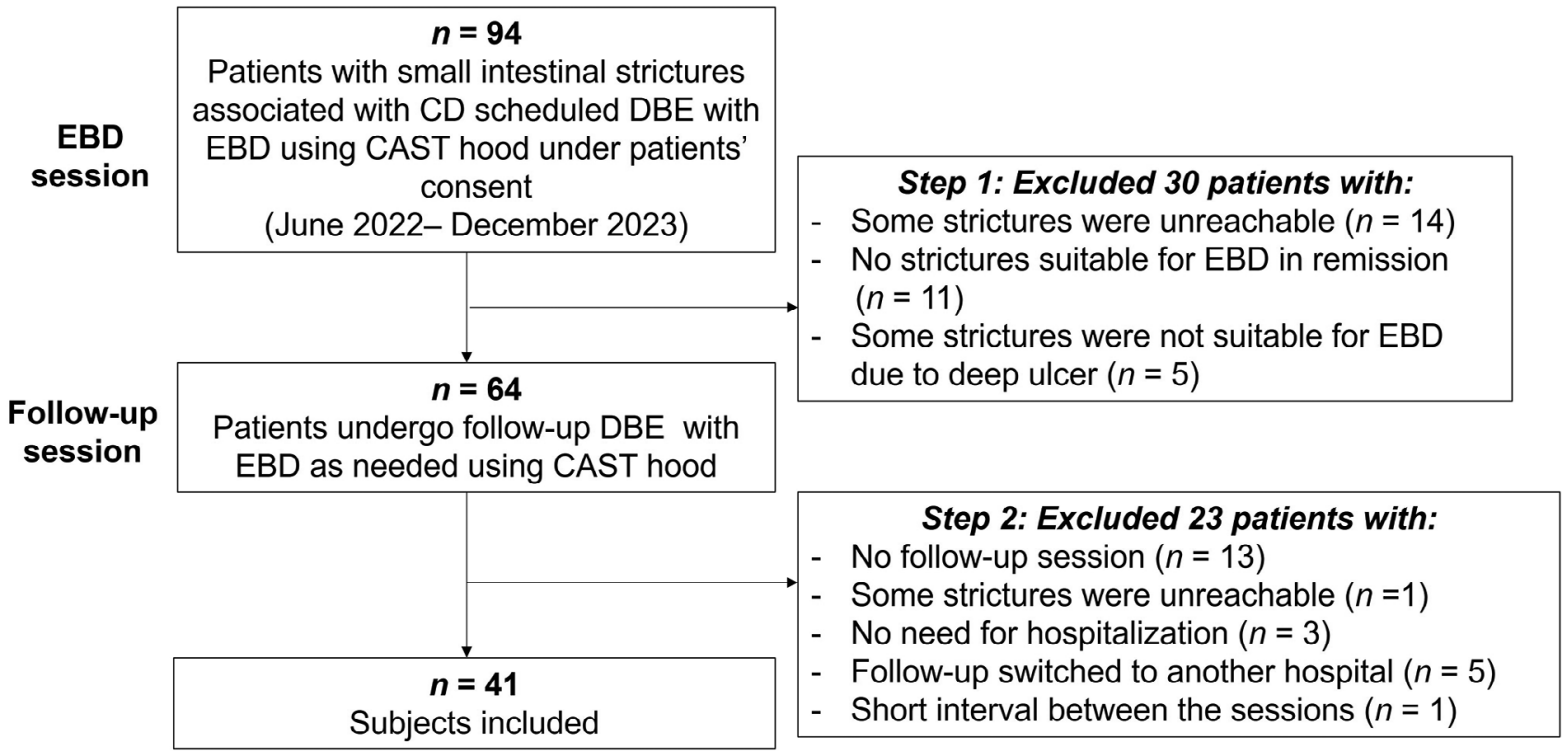
Study flowchart. Among 94 patients screened, 53 were excluded through two exclusion steps, leaving 41 patients in the study. CD: Crohn's disease, DBE: double‐balloon enteroscopy, EBD: endoscopic balloon dilation, CAST hood: calibrated small‐caliber‐tip transparent hood.

**FIGURE 3:**
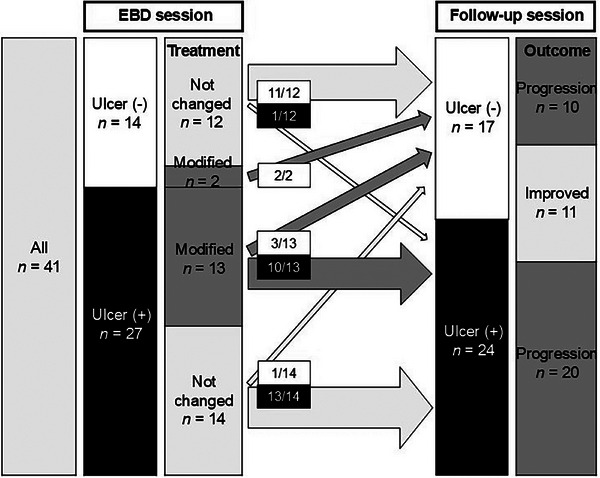
Overview of treatment modifications and clinical progress following the endoscopic balloon dilation (EBD) session.

Of 94 patients considered, 53 were excluded, leaving 41 patients (33 men; Figure 2). Table 2 summarizes their baseline characteristics. The mean interval between EBD sessions was 11.6 months. Most were asymptomatic and in serological remission, with stable abdominal symptoms and laboratory results (Table 2). At the EBD session, treatment followed Japanese IBD guidelines,^22^ with 34 (83%) receiving biologics and 25 (61%) on immunomodulators. After EBD, treatment was modified in 15 patients (37%) due to ulcers or diarrhea. These modifications included initiating biologics and/or immunomodulators in three cases and dose escalation in four cases (Table 3). Among patients with ulcers (*n* = 27), those with treatment modifications had a higher ulcer healing rate (3/13) than those who did not undergo treatment changes (1/14; Figure 3). No significant differences were observed in CDAI and SIBDQ scores between the progression and improvement groups.

**TABLE 2:** Characteristics of the study patients.

	All	Progression	Improved	
Characteristics	*n* = 41	*n* = 30	*n* = 11	*p*‐value
Gender (male: female)	33:8	24:6	9:2	1.0
Age (years), median (range)				
at diagnosis of Crohn's disease	26 (12‐58)	26 (12‐57)	43 (16‐58)	0.28
at the EBD session of the study	43.5 (19‐72)	44 (21‐69)	43 (19‐72)	0.86
Age at diagnosis, *n* (%)				
A1: below 16 years	5 (12)	4 (13)	1 (9)	1.0
A2: between 17 and 40 years	29 (71)	23 (77)	6 (55)	0.25
A3: above 40 years	7 (17)	3 (10)	4 (36)	0.069
Overall period from the initial diagnosis to the EBD session of the study, (years), mean (± SD)	13.3 ± 10.2	14.3 ± 10.0	8.4 ± 9.1	0.096
History of EBD before the study, *n* (%)	34 (83)	25 (83)	9 (82)	1.0
Period between the sessions, (months), mean (± SD)	11.6 ± 2.7	11.2 ± 2.7	12.6 ± 2.8	0.16
Patients with abdominal symptoms				
before EBD session, *n* (%)	6 (15)	6 (20)	0 (0)	0.17
before follow‐up session, *n* (%)	5 (12)	5 (17)	0 (0)	0.30
CDAI score, median (range)				
before EBD session	47 (0‐206)	51.5 (0‐206)	43 (18‐113)	0.50
before follow‐up session	42 (6‐245)	43 (6‐245)	26 (17‐85)	0.23
SIBDQ score, median (range)				
before EBD session	57 (15‐70)	56 (15‐70)	64 (49‐70)	0.10
before follow‐up session	57 (41‐70)	57 (41‐70)	60 (43‐70)	0.46
Laboratory data at EBD session, mean (± SD)				
Hemoglobin, g/dL	13.6 ± 1.5	13.5 ± 1.6	13.9 ± 1.1	0.43
Erythrocyte sedimentation rate, mm/h	6.9 ± 5.3	7.6 ± 5.3	4.4 ± 5.0	0.087
C‐reactive protein, mg/dL	0.08 ± 0.15	0.10 ± 0.17	0.03 ± 0.03	0.2
Serum albumin, g/dL	4.2 ± 0.4	4.1 ± 0.5	4.3 ± 0.2	0.28
Serum Leucine‐Rich α2 Glycoprotein, µg/mL	12.4 ± 5.9	12.9 ± 6.4	10.9 ± 4.5	0.36
Laboratory data at follow‐up session, mean (± SD)				
Hemoglobin, g/dL	13.6 ± 1.4	13.5 ± 1.5	14.0 ± 1.2	0.34
Erythrocyte sedimentation rate, mm/h	7.0 ± 7.4	7.7 ± 6.7	5.1 ± 9.4	0.33
C‐reactive protein, mg/dL	0.15 ± 0.22	0.19 ± 0.38	0.06 ± 0.07	0.27
Serum albumin, g/dL	4.1 ± 0.4	4.0 ± 0.5	4.3 ± 0.3	0.073
Serum Leucine‐Rich α2 Glycoprotein, µg/mL	12.8 ± 6.8	13.3 ± 7.4	11.4 ± 4.9	0.42

Abbreviations: CDAI, Crohn's Disease Activity Index; EBD, endoscopic balloon dilation, SD, standard deviation; SIBDQ, Short Inflammatory Bowel Disease Questionnaire.

**TABLE 3:** Medical treatments administered before and after the endoscopic balloon dilation (EBD) session.

	All	Progression	Improved	
Medical treatments	*n* = 41	*n* = 30	*n* = 11	*p*‐value
**Before the EBD session, *n* (%)**				
Elemental diet	36 (88)	25 (83)	11 (100)	0.30
5‐aminosalycilic acid	32 (78)	23 (77)	9 (82)	1.0
Steroids	2 (5)	2 (7)	0 (0)	1.0
Prednisolone	0 (0)	0 (0)	0 (0)	1.0
Budesonide	2 (5)	2 (7)	0 (0)	0.68
Immunomodulators	25 (61)	20 (67)	5 (45)	0.29
Biologics	33 (80)	25 (83)	8 (73)	0.66
Adalimumab	10 (24)	7 (23)	3 (27)	1.0
Infliximab	14 (34)	11 (37)	3 (27)	0.72
Ustekinumab	8 (20)	7 (23)	1 (9)	0.41
Vedolizumab	1 (2)	0 (0)	1 (9)	0.27
Risankizumab	0 (0)	0 (0)	0 (0)	1.0
**Treatment modified after EBD session, *n* (%)**	15 (37)	13 (43)	2 (18)	0.17
**After the EBD session, *n* (%)**				
Elemental diet	35 (85)	25 (83)	10 (91)	1.0
5‐aminosalycilic acid	32 (78)	23 (77)	9 (82)	1.0
Steroids	1 (2)	1 (3)	0 (0)	1.0
Prednisolone	0 (0)	0 (0)	0 (0)	1.0
Budesonide	1 (2)	1 (3)	0 (0)	1.0
Immunomodulators	25 (61)	21 (70)	4 (36)	0.074
Biologics	34 (83)	25 (83)	9 (82)	1.0
Adalimumab	9 (22)	6 (20)	3 (27)	0.68
Infliximab	11 (27)	9 (30)	2 (18)	0.70
Ustekinumab	10 (24)	7 (23)	3 (27)	1.0
Vedolizumab	1 (2)	0 (0)	1 (9)	0.27
Risankizumab	3 (7)	3 (10)	0 (0)	0.55

Abbreviations: EBD: endoscopic balloon dilation.

### Outcomes of EBD

During the EBD session, 159 strictures were dilated (57 ≤13.5 mm and 102 dilated to 15 mm). At follow‐up, 170 strictures were detected, with 148 (87%) dilated. Mean strictures per patient increased from 3.9 to 4.1; counts increased to 10 (24%) and decreased to nine (22%). Ulcerative strictures decreased from 66% to 59%, severe strictures decreased from 32 to 28, whereas moderate and mild strictures increased from 65 to 79 and 61 to 63, respectively. The average stricture diameter increased marginally from 9.6 to 9.7 mm, and the narrowest stricture improved in 46% of patients (from 8.0 to 8.4 mm). Overall, 30 (73%) patients experienced progression, and 11 (27%) showed improvement (Tables 2, 3, 4 and Figure 3). Univariate and multivariate analyses confirmed that ulcers in both sessions were significantly associated with progression (odds ratio 7.59; 95% confidence interval [CI] 1.20–48.10, *p* = 0.031; Table 5).

**TABLE 4:** Endoscopic findings in patients experiencing progression or improvement following endoscopic balloon dilation (EBD).

	All	Progression	Improved	
Endoscopic findings	*n* = 41	*n* = 30	*n* = 11	*p*‐value
Number of strictures per patient in the EBD session, *n*	3.9 ± 2.9	3.7 ± 2.8	4.4 ± 3.3	0.52
Presence of ulcers				
In the EBD session, *n*	28 (68)	23 (77)	5 (45)	0.073
In the follow‐up session, *n*	24 (59)	20 (67)	4 (36)	0.15
In both the EBD session and the follow‐up session, *n*	23 (56)	20 (67)	3 (27)	**0.036**
Mean diameter of all strictures in each patient in the EBD session, mm	9.2 ± 2.1	9.2 ± 2.0	9.3 ± 2.3	0.87
Mean diameter of the narrowest stricture in each patient in the EBD session, mm	8.0 ± 2.2	8.2 ± 2.2	7.4 ± 2.1	0.30
Mean diameter of the dilation balloon for the narrowest stricture in the EBD session, mm	13.9 ± 1.3	13.8 ± 1.3	14.3 ± 1.4	0.27
Dilation balloon ≥15 mm for the narrowest stricture in the EBD session, *n*	21 (51)	14 (47)	7 (64)	0.48

Data are presented as *n* (%) or mean ± standard deviation, EBD; endoscopic balloon dilation.

**TABLE 5:** Univariate and multivariate analyses of factors contributing to progression.

	Univariate analysis			Multivariate analysis		
Factors	OR	95% CI	*p*‐value	OR	95% CI	*p*‐value
Gender (male)	1.12	0.16–13.40	1.0	1.17	0.11–13.00	0.90
Age at diagnosis of Crohn's disease	0.97	0.91–1.03	0.28	1.14	0.88–1.47	0.31
Age at the EBD session of the study	0.96	0.94–1.05	0.86	0.84	0.65–1.08	0.18
Overall period from the initial diagnosis to the EBD session of the study, years	1.08	0.99–1.18	0.10	1.22	0.95–1.57	0.11
History of EBD before the study, (>1 time)	0.90	0.12–11.10	1.0			
Period between the sessions, months	0.79	0.57–1.10	0.17			
Laboratory data in the EBD session						
Hemoglobin	0.81	0.49–1.35	0.42			
Erythrocyte sedimentation rate	1.15	0.99–1.36	0.097			
C‐reactive protein	–	0.00047–inf	0.19			
Serum albumin	0.32	0.042–2.460	0.28			
Serum leucine‐rich α2 glycoprotein	1.07	0.93–1.24	0.36			
Laboratory data in the follow‐up session						
Hemoglobin	0.76	0.44–1.32	0.33			
Erythrocyte sedimentation rate	1.06	0.94–1.19	0.33			
C‐reactive protein	54.1	0.022–134,000	0.32			
Serum albumin	0.164	0.022–1.25	0.081			
Serum Leucine‐Rich α2 Glycoprotein	1.06	0.93–1.21	0.42			
Medical treatments in the EBD session (yes/no)^*^						
Elemental diet	–	0.34–inf	0.30			
5‐aminosalycilic acid	1.36	0.20–15.80	1.0			
Steroids	0	0–106.1	1.0			
Immunomodulators	0.25	0.04–1.30	0.074			
Biologics	0.90	0.12–11.10	1.0			
Treatment modification after EBD session (yes/no)^*^	0.30	0.03–1.82	0.17			
Endoscopic findings						
Multiple strictures in the EBD session (yes/no)^*^	0.28	0.0056–2.6300	0.40			
Number of strictures per patient in the EBD session	0.93	0.73–1.17	0.52			
Presence of an ulcer						
In the EBD session (yes/no)^*^	3.79	0.72–21.57	0.073			
In the follow‐up session (yes/no)^*^	3.39	0.67–19.84	0.15			
In both the EBD session and the follow‐up session (yes/no)^*^	5.1	0.96–36.52	0.036	7.59	1.20–48.10	0.031
Mean diameter of all strictures in each patient in the EBD session	0.97	0.70–1.36	0.87			
Diameter of the narrowest stricture in each patient in the EBD session	1.21	0.85–1.73	0.30			
Presence of severe stricture in the EBD (diameter ≤ 7 mm) (yes/no)^*^	0.64	0.13–3.18	0.73			
Dilation balloon ≥15 mm for the narrowest stricture in the EBD session	0.51	0.089–2.160	0.48			

Abbreviations: CI, confidence interval; EBD, endoscopic balloon dilation; OR, odds ratio.

* Refference = no.

Patients with 15 mm balloons for their narrowest strictures showed better improvement than those treated with smaller balloons. More patients with ulcerative strictures experienced progression during EBD (68%) and follow‐up (59%) sessions. Compared with patients in the improved group, those in the progression group had a significantly higher incidence of ulcerative strictures in both sessions (67% vs. 27%; *p* = 0.036; Table 4).

**FIGURE 4:**
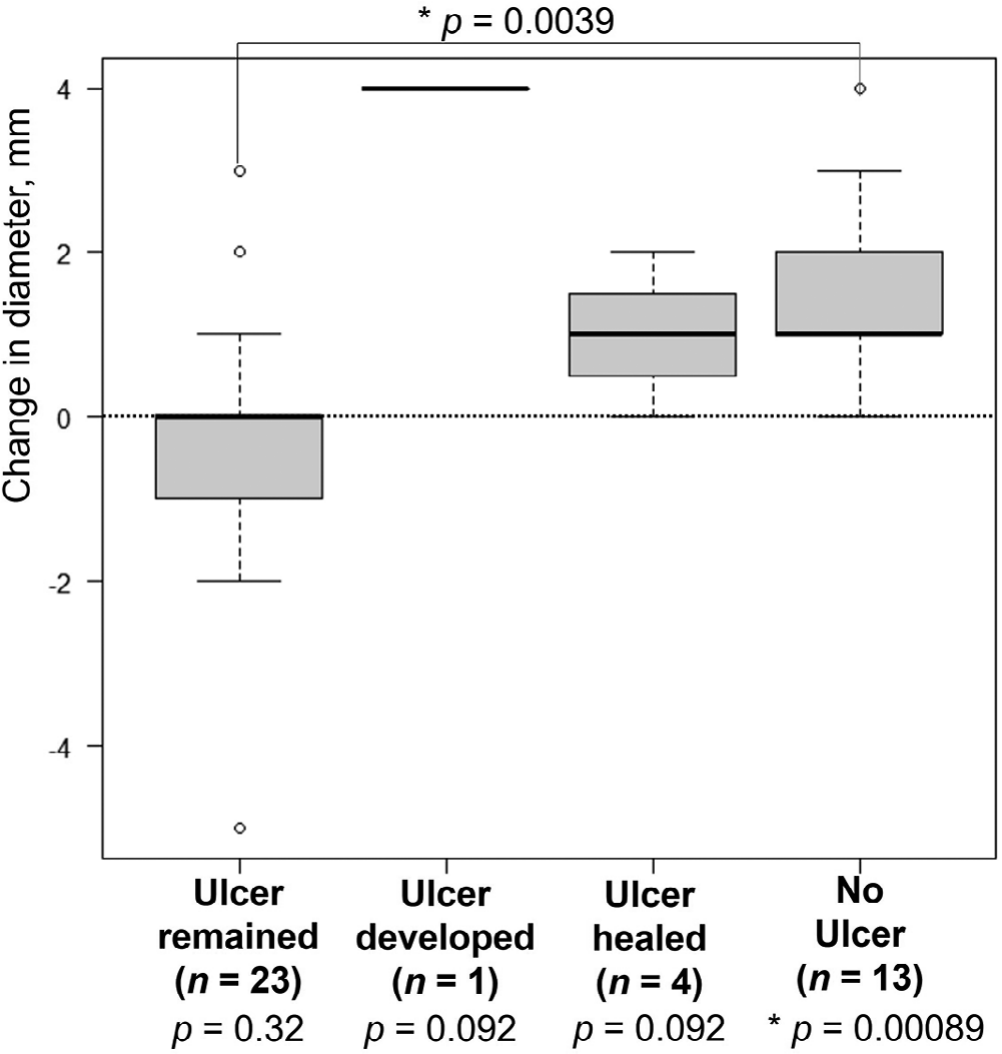
Variations in the diameter of the narrowest stricture. Bars indicate the mean, with error bars representing the standard deviation. An asterisk denotes a *p*‐value < 0.05 (Paired t‐test and Tukey's test).

Given the association between ulceration and progression, patients were categorized into four groups based on the presence of ulcers across sessions: ulcer‐remaining (*n* = 23), ulcer‐developed (*n* = 1), ulcer‐healed (*n* = 4), and no ulcer (*n* = 13; Figure 3). The no‐ulcer group showed significant improvement in the narrowest stricture diameter (from 9.0 to 10.5 mm, +1.5 mm, *p* = 0.00089; Figure 4). Improvement was significantly greater in the no‐ulcer group compared with the ulcer‐remaining group (+1.5 mm vs. ‐0.4 mm, *p* = 0.0039; Figure 4). Progression rates were highest in the ulcer‐remaining group (87%, *n* = 20), whereas the ulcer‐developed (0%, *n* = 0), ulcer‐healed (75%, *n* = 3), and no‐ulcer (54%, *n* = 7) groups had lower rates.

### Procedure‐related adverse event

Overall, 99 DBE procedures were performed across both sessions, with only one reported adverse event (intestinal perforation at the EBD site), which occurred during the follow‐up session after EBD. The patient required emergency surgery and subsequently recovered. No cases of post‐procedural bleeding or acute pancreatitis were reported.

### Biomarker accuracy for predicting mucosal condition

**FIGURE 5:**
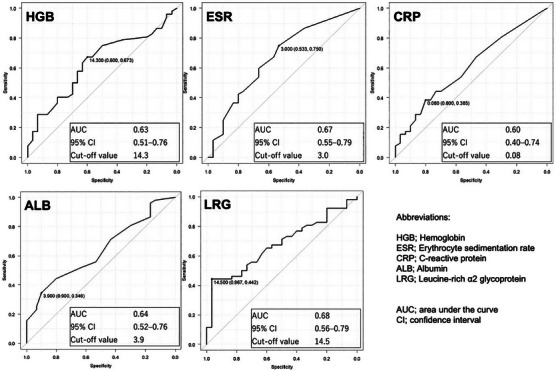
Receiver operating characteristic analysis of hemoglobin, erythrocyte sedimentation rate, C‐reactive protein, serum albumin, and serum leucine‐rich α2 glycoprotein for accurately predicting the mucosal condition of the small intestine.

During the study period, 82 sessions (41 patients underwent two sessions) were performed. Each session was divided into two groups, with or without an ulcer, to evaluate the accuracy of each biomarker in predicting the mucosal condition. The mean hemoglobin (HGB), erythrocyte sedimentation rate (ESR), C‐reactive protein (CRP), serum albumin (ALB), and serum LRG were 13.6 g/dL, 6.9 mm/h, 0.23 mg/dL, 4.1 g/dL, and 12.6 µg/mL, respectively. Among them, HGB and ALB were significantly lower, and LRG was significantly higher in sessions with an ulcer (Table 6). Furthermore, ROC curve analysis showed that the AUC of HGB, ESR, CRP, ALB, and LRG were 0.63, 0.67, 0.60, 0.64, and 0.68, respectively, with LRG being the highest among the five biomarkers (Figure 5).

**TABLE 6:** Biomarkers in sessions with and without ulcers.

		Ulcer		
	All	(+)	(‐)	
Biomarkers	*n* = 82	*n* = 51	*n* = 31	*p*‐value
Laboratory data at each session, mean (± SD)				
Hemoglobin (HGB), g/dL	13.6 ± 1.4	13.4 ± 1.5	14.1 ± 1.2	0.043
Erythrocyte sedimentation rate (ESR), mm/h	6.9 ± 6.4	7.8 ± 6.2	5.2 ± 6.7	0.077
C‐reactive protein (CRP), mg/dL	0.12 ± 0.26	0.15 ± 0.32	0.06 ± 0.09	0.12
Serum albumin (ALB), g/dL	4.1 ± 0.4	4.1 ± 0.5	4.3 ± 0.3	**0.017**
Serum leucine‐rich α2 glycoprotein (LRG), µg/mL	12.6 ± 6.4	14.1 ± 7.3	10.0 ± 3.0	**0.0044**
Meeting strict criteria at each session, *n* (%) (HGB > 13, ESR < 5, CRP < 0.1, ALB > 4.0, and LRG < 8.0)	9(11)	6(12)	3(10)	1.0

Data are presented as *n* (%) or mean ± standard deviation.

However, an ulcer could not be ruled out even when all biomarkers were within normal limits. The rate of meeting strict criteria (HGB > 13, ESR < 5, CRP < 0.1, ALB > 4.0, and LRG < 8.0) was not significantly different between the session with an ulcer and the session without an ulcer (*p* = 1.0; Table 6).

## DISCUSSION

This prospective study is the first to evaluate the efficacy of EBD for CD‐associated small intestinal strictures, specifically examining factors affecting progression through stricture diameter measurements during DBE. This study prospectively identified the key factors associated with the recurrence of strictures following a single EBD session.

The total number of strictures increased, likely because of persistent ulcers. However, this does not imply that EBD is ineffective. The number of severe strictures decreased; however, moderate and mild strictures increased, and the average stricture diameter slightly expanded. These observations suggest that EBD when combined with optimized treatment based on endoscopic findings, facilitates better assessment of the small intestine and allows for earlier intervention in managing strictures.

EBD is a well‐established, minimally invasive treatment for managing CD‐related small intestinal strictures. Despite numerous studies reporting positive long‐term outcomes, repeat dilation is frequently required.^12^, ^23^, ^24^, ^25^, ^26^ Owing to the challenges in accurately measuring stricture diameters, few studies have explored restenosis and diameter changes after EBD.

Previous studies have highlighted the high rate of restenosis requiring repeated EBD, prompting the introduction of a novel definition of progression. Direct comparison of each stricture was challenging in 78% of the patients with multiple strictures. Therefore, progression was defined based on changes in the narrowest stricture diameter. In this study, 73% of patients experienced progression, which was significantly associated with the presence of ulcers during EBD and follow‐up sessions. This high rate aligns with prior studies using different criteria,^9^ but does not suggest that EBD is ineffective. These data indicate that repeated scheduled DBE with maintenance EBD could contribute to suppressing stricture progression and enable endoscopic stricture management. No significant differences were observed, although the improved group had a narrower stricture diameter than that of the progression group. However, the EBD diameter tended to be larger, with most dilations reaching 15 mm. Patients who achieved complete mucosal healing demonstrated substantial improvement in the narrowest stricture diameter following EBD.

Long‐term EBD outcomes are measured by symptom‐free and surgery‐free rates,^10^, ^11^, ^12^ yet factors such as diet, adherence, and personal or physician preferences complicate identifying restenosis risks. Hibiya et al. reported that ulcers at stricture sites increase the risk of repeated dilation and surgery (hazard ratio 4.84, 95% CI 1.58–14.79).^27^ In our study, even small ulcers affected EBD outcomes, with complete mucosal healing improving stricture diameter (+1.5 mm), highlighting its importance.

Schulberg et al. recommended combining anti‐inflammatory therapy with endoscopic treatment for strictures.^28^ Our results support an optimized post‐EBD strategy, guided by endoscopic findings and a treatment‐to‐target strategy, to sustain mucosal healing.

Although the CDAI and SIBDQ assess CD activity and quality of life, they did not correlate with endoscopic findings in our study. LRG, a novel serum biomarker induced by interleukin (IL)‐22, TNF‐α, and IL‐1β independent of IL‐6, is upregulated in CD and correlates better with CDAI than CRP.^29^ Although some studies suggested that LRG detected small bowel mucosal activity in patients with CD,^30^, ^31^, ^32^ the evidence remains limited. Our findings show that LRG has superior diagnostic value for identifying small intestinal ulcers, with a sensitivity of 0.967 and specificity of 0.442. HGB, ALB, and LRG were significantly linked to ulcer presence, with LRG achieving the highest AUC. However, normal biomarker levels do not exclude ulcers, so endoscopic evaluation remains essential. Annual DBE with maintenance EBD is a reasonable monitoring strategy.

This study has some limitations, including its single‐center design and small sample size, which limit the statistical analysis and the generalizability of the findings. Additionally, comparing stricture diameters on a one‐to‐one basis was complicated by the presence of multiple and newly developed strictures. The analysis focused on the narrowest stricture in each patient, as these strictures often cause symptoms and tend to change less between sessions.

In conclusion, achieving and maintaining complete mucosal healing is critical for preventing progression following EBD in patients with CD‐induced small intestinal strictures.

## CONFLICT OF INTEREST STATEMENT

Hironori Yamamoto holds patents related to the calibrated small‐caliber‐tip transparent hood and double‐balloon enteroscopy and has an association with Fujifilm. Tomonori Yano received research funding and honoraria from Fujifilm, Japan. Hirotsugu Sakamoto received research funding from Fujifilm and scholarship donations from Fujifilm Medical Co., Ltd. The authors declare no personal financial ties to any commercial entity involved in the production of healthcare‐related products or services pertinent to this study.

## ETHICS STATEMENT

The Institutional Ethical Review Board of Jichi Medical University (the board approval number: 21–141) approved this study, which was conducted in accordance with the Declaration of Helsinki.

## PATIENT CONSENT STATEMENT

Written informed consent was obtained from the participants in this study.

## CLINICAL TRIAL REGISTRATION

This study was registered with the Japan Registry of Clinical Trials (jRCT) under the registration number jRCT1030220093.

## Data Availability

The data supporting the findings of this study are not publicly available due to privacy concerns regarding research participants. However, they can be obtained from the first author, Jun Owada, upon reasonable request.
